# Automatic multi-cycle analysis of cardiac function from real-time MRI

**DOI:** 10.1186/1532-429X-17-S1-P385

**Published:** 2015-02-03

**Authors:** Teodora Chitiboi, Anja Hennemuth, Lennart Tautz, Markus Huellebrand, Jens Frahm, Horst Hahn

**Affiliations:** 1 Fraunhofer MEVIS, Bremen, Germany; 2 Max Planck Institute for Biophysical Chemistry, Goettingen, Germany

## Background

MR imaging plays a crucial role in the early detection, monitoring and treatment planning of cardiovascular disease, which is the leading cause of death in the Western countries. A recently developed real-time MRI technique allows for the inspection and visualization the heart muscle (myocardium) dynamics with high temporal resolution (up to 20ms) under free breathing and without the need for ECG synchronization. However, the quantification of cardiac function becomes more challenging than for conventional cardiac imaging methods because of the extremely large amount of data (several hundred time frames per image slice, spanning over 10-20 or more consecutive cardiac cycles), as well as potential changes in contrast properties and respiratory displacements. In order to overcome such problems, we developed a new method for automatic analysis of cardiac function from real-time MRI.

## Methods

Real-time cardiac MRI in short-axis orientation was performed in four healthy subjects (mean age 25 years) and one patient with arrhythmia using a Siemens Trio 3T scanner. Acquisition is based on a highly undersampled radial FLASH sequence with and without a bipolar flow-encoding gradient and image reconstruction by regularized nonlinear inversion. The spatial resolution was 1.6x1.6x6.0mm and the temporal resolution of to 30 ms.

The image series were analyzed in Fourier domain to separate breathing motion from heart motion. The myocardium was segmented fully automatically in all time frames using a context based approach. After segmentation, the cardiac cycles are automatically determined based on the local extrema in the blood volume time curves.

## Results

The results of the automatic segmentation were compared to the manual results of two experts, obtaining an average dice score of 95.4% for the epicardium and 89.8% for the endocardium.

Plotting the blood volume for each image series over time enables the visualization of 10-15 consecutive heart cycles for each individual heart slice and the analysis of inter-cycle variations of functional parameters. The effects of breathing, physiologic maneuvers, stress, and arrhythmia, which are inaccessible by regular cine MRI, can also be observed in the plot.

Automatic quantification of cardiac function from real-time MRI enables analysis of patients with irregular heartbeats (e.g. arrhythmias), is undisturbed by free breathing and enables the monitoring of cardiac dynamics responses to stress or physiologic maneuvers.

## Conclusions

The manual and automatic myocardium segmentation shows good agreement. Because no manual input is required, the proposed method is suited for the automatic analysis of cardiac function in real-time MRI, which could previously not be achieved automatically or with reasonable amount of human effort.

## Funding

Fraunhofer and Max-Planck Society.

**Figure 1:**
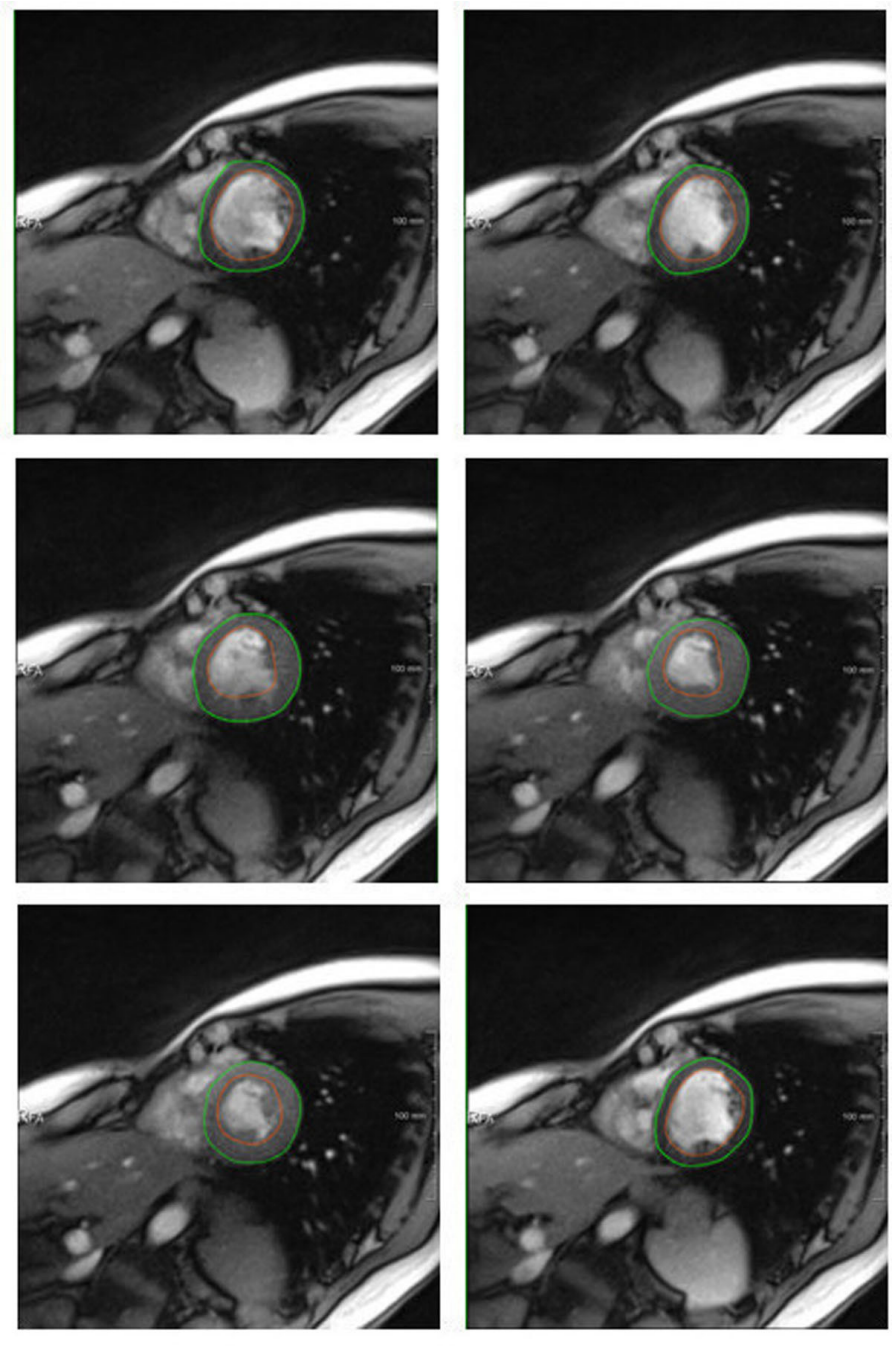
Sample fully automatic myocardium segmentation results.

**Figure 2:**
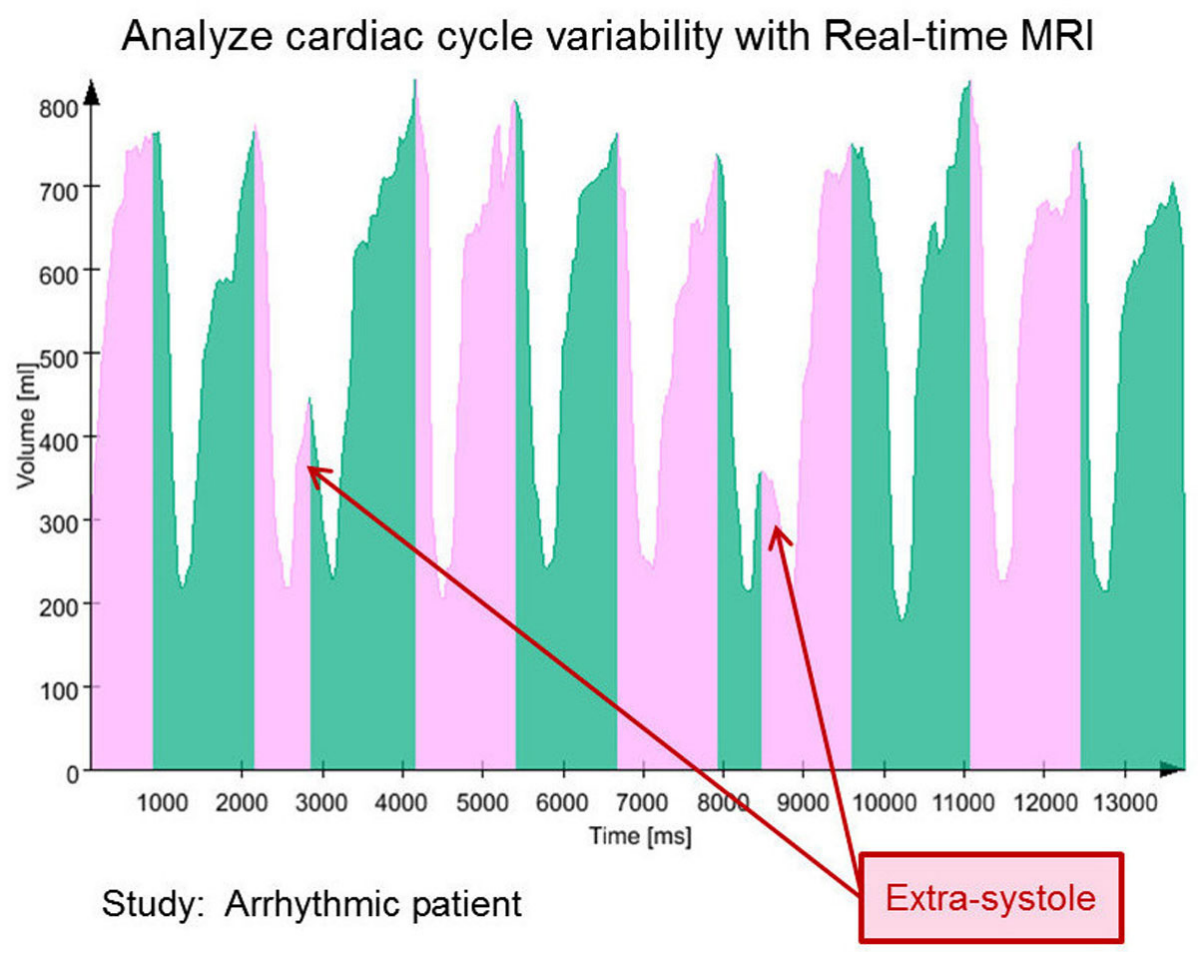
Blood volume over time for twelve consecutive cardiac cycles (in alternatic colours) for a mid-ventricular short axis slice. The patient presents axtra-systole caused by arrhythmia which result in smaller end-diastolic volume.

